# Are physical therapists in Viet Nam ready to implement evidence-based practice? A survey

**DOI:** 10.1186/s12909-018-1428-3

**Published:** 2018-12-22

**Authors:** Hiep Thi Dao, Sopa Pichaiyongwongdee, Patricia E. Sullivan, Saipin Prasertsukdee, Benjawan Apinonkul

**Affiliations:** Faculty of Physical Therapy, Mahidol University, 999 Phuttamonthon 4 Road, Salaya, Nakhon Pathom, 73170 Thailand

**Keywords:** Barriers, Evidence-based practice use, Physical therapy, Viet Nam

## Abstract

**Background:**

Evidence-based practice (EBP) enhances healthcare services and keeps providers current with best practices. EBP has been adopted and spread worldwide. However, people will not apply it if they do not know, understand, or believe it. Few studies have considered EBP application in Viet Nam. This study explores whether Vietnamese physical therapists’ attitude, knowledge, skills toward EBP and barriers to its use make them ready to implement its practice.

**Methods:**

A survey questionnaire was sent directly to physical therapists in governmental healthcare organizations in Ho Chi Minh City, Viet Nam, from July to October, 2017. It consisted of 41 closed- and open-ended questions related to knowledge, attitude, behaviors, frequency of use, and barriers of EBP and the demographic characteristics of participants. Descriptive statistics and significant correlations were determined from Chi-Square statistics or odds ratios between the variables.

**Results:**

The return rate was 93% (421 out of 453). Eliminated were 40 responses inconsistent with inclusion criteria. The 381 eligible participants were more female (62%) than male, about 53% had vocational degrees, less than 1% had M.S. degrees. Participants reported a positive attitude toward EBP. An incongruity existed between knowledge/ skills of EBP and the frequency of using its 5 steps. English competence was the most critical barrier to applying EBP. The significant associations between attitude and knowledge, and demographical attributes indicated that younger therapists with lower educational degrees had less knowledge of EBP and they rarely employed the application and analytical steps 4 and 5.

**Conclusions:**

The incongruity between knowledge and use of EBP may result from the lack of EBP in academic education. The skills of reading professional articles in the English language and understanding and applying the steps of EBP should be emphasized in academic physical therapy programs. Additionally, policy makers should consider the number of patients a day per physical therapist which impacts EBP use and the quality of healthcare service.

**Electronic supplementary material:**

The online version of this article (10.1186/s12909-018-1428-3) contains supplementary material, which is available to authorized users.

## Background

Evidence-based practice (EBP) is the combination of knowing and applying the best evidence, understanding patients’ preference, and utilizing clinical expertise to optimize patient care and to facilitate the clinical decision-making (CDM) of healthcare providers [1]. Internationally, EBP is included in healthcare professional education as a compulsory competency and as a part of the core academic competencies across disciplines [2–4]. The commonly reported5 steps of EBP are: (i) converting clinical queries into answerable questions; (ii) finding the best evidence to answer those questions; (iii) critically appraising the evidence; (iv) integrating the best evidence with the patient’s preference and the practitioners clinical expertise to make clinical decisions; and (v) evaluating the effectiveness of the clinical practice change from the evidence used [5]. Employing these 5 steps is a complicated process. It is influenced by individual and environmental factors [6] and it requires individual and organizational efforts [7, 8]. Practitioners need to know the process of EBP, have time, and resources to search the literature, and have the administrative support to implement changes in practice. Lack of knowledge of EBP, especially understanding and interpreting statistics, is a barrier consistently found among healthcare providers across countries [9–12]. Scurlock-Evan et al. [13] reported that the majority of physical therapists (PTs) have a positive attitude toward EBP, but there is low use. Common barriers include lack of time, due to the number of patients seen per day, poor searching and statistical skill, and misunderstanding of EBP. In addition, English reading competency is reported to be a barrier in countries where English is not the first language [14, 15].

In developing countries, the implementation of EBP needs to be expanded in order to improve patient outcome [9, 16, 17]. Melnyl et al. [18] suggested that identifying and understanding potential barriers to EBP are the initial steps to develop strategies toward successful implementation. One step toward implementing EBP is to require research methodology, one part of EBP process, as a standard educational competency for Vietnamese healthcare providers [19–21]. However, EBP is more than understanding the research process and statistical implication. The critical analysis, clinical application, and evaluation aspects of EBP are essential to know and apply in order for implementation. Facilitators and barriers have been reported in Vietnamese nurses and physicians. They reported their lack of knowledge of EBP and lack of confidence in applying EBP. Some of these health professionals were even not familiar with the EBP term [10, 22].What is not known is the prevalence of knowledge and practice of EBP among currently practicing physical therapists in Viet Nam.

Currently, there are 3 public and 1 private universities running university level physical therapy programs in Viet Nam. In contrast to this university education, there are 2-year certificate or diploma training programs, approximately 15 upper secondary vocational schools, general colleges, or medical colleges running a 2-year certificate, or 3-year B.Sc. for physical therapy programs in the south of Viet Nam. Entry level Master’s Degree for physical therapy does not exist in Viet Nam. After graduation, therapists work under the supervision of doctors of medicine, traditional medicine, or rehabilitation physicians in hospitals, rehabilitation centers, nursing and rehabilitation hospitals, or universities. These institutes are regulated by different governmental agencies that provide health care to approximate 8.6 million inhabitants of Ho Chi Minh City (HCMC), which is the biggest city in Viet Nam. Physical therapy practice is based on physician prescriptions and PTs decide specific methods to apply on their patients, which may or may not be optimal for patients’ conditions. The need to improve patient outcomes is necessary. An initial step to optimize outcomes is to determine if physical therapists in Viet Nam are ready to implement EBP and if not what might need to change. To that end, we explored factors related to the current use of EBP, including attitudes, knowledge, behaviors, and perceived barriers interrelated with the demographic characteristics of therapists. The findings will help direct effective continuing education, and academic programs to introduce, and educate physical therapists for EBP in Viet Nam and help to overcome barriers.

## Methods

### Study design, sample

A cross-sectional survey used a self-administered questionnaire to explore the physical therapists’ attitudes, knowledge, behaviors, and perceived barriers toward EBP. This study was approved by the Central Institutional Review Board of Mahidol University in Thailand and supported by the HCMC Health Department. A sampling frame was created using the lists of 453 PTs’ phone numbers and email addresses supplied by 54 out of 60 organizations in HCMC having PT units. The organizations were regulated by one of the governmental healthcare authorities.

### Procedure

The 41 item questionnaire integrated those developed by Jette [23] and Upton [24] and queried the 5 steps of EBP. Eleven questions inquired about the attitudes toward the use, limitation, and perceived benefit toward EBP; 8 questions were about the knowledge/skills relating to learning the 5 steps of EBP, and English reading ability; 6 questions were about the frequency of EBP use, 3 questions about the use of EBP. A ranking question was about evidence sources most frequently used and the three top barriers impacting the use of EBP; 11 questions were about demographic characteristics. A space was provided for information to be added.

The questionnaire’s content was validated by four experts who have more than 10 years of experience in EBP and are teaching and applying research in the United States of America (USA) or in Thailand’s universities. They reviewed the survey questions to ensure that the developed questions complied with their expert opinions and met the study objectives. Once approved, the questions were translated into Vietnamese by two native speakers, one teaching English at the University of Medicine and Pharmacy for more than 20 years, and the other a university professor living in Canberra, Australia. Once agreed, the synthesized questionnaire was generated.

To validate suitability, the questions were trialed with 10 people from different healthcare fields: clinical instructors, nurses, and PTs. The questions were then slightly rephrased and modified to remove potential ambiguity. This developed questionnaire took an average of 18 min to complete. The questionnaire was added as an Additional file 1.

After receiving permission from the directors of the governmental organizations, the researcher contacted the chiefs of the PT units to discuss the process and seek cooperation. A cover letter provided important information about the study, the potential contributions of this study to communities, and an explicit statement on the voluntary and confidential nature of participants, informed consents, and the coded questionnaires, were delivered directly by the research assistants or indirectly by the chiefs of PT units to the participants. Participants with less than 1 year of experience or did not have a PT degree were excluded. The questionnaires were collected after two weeks. All consent forms were obtained from the participants who agreed to join the survey. The survey was conducted in HCMC from June to December, 2017.

### Data analysis

The survey data were analyzed using SPSS, version 20, using descriptive statistics to summarize the responses and Pearson Chi-Square or odds ratios to determine the associations between ages, years of experience, qualifications, and number of patients a day; and EBP-related responses such as attitude, knowledge, and frequency of EBP implementation. For questions that employed a 5-point Likert scale, the responses were collapsed to allow for general comparisons with other international studies. For example: *agree* includes agree and strongly agree; *disagree* includes neutral, disagree, and strongly disagree [23, 25]. As some subsamples of demographic data were small, we collapsed them together to ensure the correlation assumptions. The education qualification was categorized into vocational/ 3-year B.Sc. and 4-year B.Sc. degrees. When associations were found, were performed additional tests (odd ratio) to observe the trends among groups.

## Results

### Demographic characteristics

The return rate was 93% (421 out of 453). Only 381 questionnaires were analyzed, because some respondents did not meet the inclusion criteria. Participants were more females (62%) than males (38%); mostly in two age ranges: 20–29 (36%) and 30–39 (42%). Years of experience were roughly equally distributed among the 4 age groups. About 75% of participants earned certificates as their entry levels for PT. Some therapists (22%), who initially acquired certificates, continued their education at university programs. This brought the analyzed number of participants who held certificates to 53%. Only 1% of participants had M.Sc. as their highest degrees, so these persons were analyzed with the 4-year B.Sc. group. Most (83%) participants had not conducted any research. Most (69%) participants had more than 11 patients a day (Table 1).

**Table 1:** Demographic characteristics

	N	%
Sex		
Male	146	38.3
Female	235	61.7
Age (years)		
20–29	138	36.2
30–39	159	41.7
40–49	61	14
50 and up	23	6.0
Years of experience		
≤ 1–5	116	30.4
6–9	88	23.1
10–14	79	20.7
15 up	98	25.7
Highest degree for PT		
Vocational school and college	209	54.9
Bachelor	169	44.4
Master and Ph.D.	3	.8
Entry level for PT		
Vocational high school	284	74.5
College	7	1.8
Bachelor	89	23.4
Master	1	.3
Being clinical instructor		
Yes	99	26
No	282	74
Number of years to be instructor (years)		
0	285	74.8
1 to 23	96	25.2
Number of research		
0	315	82.7
1–3	61	16
4–10	5	1.3
Number of patients daily		
1–3	12	3.1
4–6	21	5.5
7–10	85	22.3
11+	263	69
Number of hours for taking patients		
≤ 10	23	6.0
11–20	12	3.1
21–30	27	7.1
31–39	140	36.7
≥ 40	179	47

### Attitudes toward EBP

Between 80 and 90% of participants agreed that EBP is necessary and useful, or that EBP improves patient care and helps practitioners remain current with healthcare trends. When asked if EBP does not place unreasonable demands on their duties, 70% of participants agreed. However, 40% of participants recorded neutral answers to the two statements that evidence is not enough to support their practice and that EBP does not take into account patient’s preference (Table 2).

**Table 2:** Attitudes of Viet Nam PTs toward EBP

No.	Attitudes toward, use of, perceived benefits and limitations of EBP	Disagree		Agree	
		N	%	N	%
1	Application of EBP is necessary for my practice.	32	8.4	348	91.6
2	Literature and research findings are useful in my day-to-day practice.	54	14.2	327	85.8
3	I need to increase the use of evidence in my daily practice.	70	18.4	311	81.6
4	The adoption of EBP places an unreasonable demand on physical therapists.	280	73.5	101	26.5
5	I am interested in learning or improving the skills necessary to incorporate EBP into my practice.	34	8.9	347	91.1
6	EBP improves the quality of patient care.	39	10.2	342	89.8
7	EBP does not take into account the limitation of my clinical practice setting.	309	81.1	72	18.9
8	EBP helps clinicians remain up-to-date with healthcare trends.	53	13.9	328	86.1
9	Strong evidence is lacking to support most of the interventions I use with my patients.	243	63.8	138	36.2
10	EBP helps me make decisions about patient care.	69	18.1	312	81.9
11	EBP does not take into account patient preference.	222	58.3	159	41.7

Only half (49%) of participants reported confidence in applying Patient-Intervention-Comparison-Outcome (PICO) formatted questions. Only 25% agreed that their English language skills were adequate for understanding research articles. Neutral responses were chosen by approximately 30% for converting questions into PICO, searching for scientific evidence, and appraising professional literature (Table 3). In contrast, a large percentage (80%) of participants reported being aware of information types and resources, and being able to apply or review their own work after applying the found evidence. Slightly more than 60% agreed that they were able to search for evidence and critique professional articles. About half of the participants indicated that they learned EBP principles or research methodology in their education or self-study.

**Table 3:** Knowledge of 5 steps of EBP

No.	Knowledge/ Skill of EBP	Disagree		Agree	
		N	%	N	%
12	I am confident in converting information needed into formatted questions using PICO formulation.	196	51.4	185	48.6
13	I am aware of major information types and resources.	83	21.8	298	78.2
14	I am able to search for the scientific evidence relevant to the formulated question.	147	38.6	234	61.4
15	I am confident in my ability to critically appraise professional literature.	140	36.7	241	63.3
16	I am able to apply evidence to my own cases.	88	231	293	76.9
17	I am able to review my own work after applying the found evidence.	81	21.3	300	78.7
18	I learned the foundation for EBP as part of my vocational/ tertiary education/ continuing education courses/ self-taught education.	183	48	198	52
19	I received Research Methodology as part of my vocational/ tertiary education/ continuing education courses/ self-taught education.	207	54.3	174	45.7
20	My English language skills are adequate for understanding research articles.	285	74.8	96	25.2

### Frequency of 5 steps of EBP use

Between 40 to 48% of the participants reported that they used EBP steps at least monthly; whereas, 42% of participants did not convert needed information into PICO questions or obtain relevant evidence. We collapsed the never or monthly responses: almost 70% did not identify the strength and weakness of found evidence; integrate evidence with clinical expertise 60%, evaluate the outcomes after applying evidence 57%, or share ideas and information with colleagues about the outcome almost 60%. Similarly, there was a low frequency of daily, weekly, or 2-week use of the EBP steps: step 1 asking answerable questions (3.9–7.3%) or step 2 finding the evidence (1.3–7.1%) (Table 4).

**Table 4:** Frequency of EBP use

No.	Frequency of EBP use (over 6 months)	Never	Monthly	Every fortnight	Weekly	Daily
21	How often have you converted information needed into formatted questions by using PICO?	156 (40.9)	161 (42.3)	21 (5.5)	28 (7.3)	15 (3.9)
22	How often have you obtained the evidence relevant to your formulated questions?	160 (42.0)	167 (43.8)	27 (7.1)	22 (5.8)	5 (1.3)
23	How often have you identified the strength and weakness of evidence you found?	101 (26.5)	164 (43.0)	52 (13.6)	44 (11.5)	20 (5.2)
24	How often have you integrated the found evidence with your expertise for making clinical decisions?	46 (12.1)	182 (47.8)	49 (12.9)	51 (13.4)	53 (13.9)
25	How often have you evaluated the outcome of your applying evidence in your practice?	56 (14.7)	163 (42.8)	66 (17.3)	53 (13.9)	43 (11.3)
26	How often have you shared your ideas and information with colleagues about the outcome of applying evidence?	44 (11.5)	182 (47.8)	45 (11.8)	70 (18.4)	40 (10.5)

### Attention to EBP use

Fewer than 2 articles were read per month by 41%; from 2 to 5 articles by 37%. Professional literature and research findings were used in the process of making clinical decisions by 42.5%. Almost half of the participants used Medline or other databases to search for practice-relevant research fewer than 2 times in a typical month (Table 5).

**Table 5:** Attention to EBP use

No.		≤ 1 article		2–5 articles		≥ 6 articles	
		N	%	N	%	N	%
27	I read/ review research/ literature related to my clinical practice.	156	40.9	141	37.0	84	22.0
		≤ 1 time		2–5 times		≥ 6 times	
28	I use professional literature and research findings in the process of clinical decision-making.	107	28.1	162	42.5	112	29.4
29	I use Medline or other databases to search for practice-relevant literature/research.	185	48.6	107	28.1	89	23.4

### Source used for CDM

Most participants relied on personal experience (96%), peers (93%), and textbooks (86%) when making clinical decisions. They also used doctors and discussions with colleagues as other sources in their CDM.

### Barriers toward EBP

The top three barriers to implement were: reading research literature written in foreign languages (84%); insufficient time (72%), and limited access to sources of information (73%). Some participants identified additional barriers: shortage of equipment, knowledge of how to apply to patients, limited literature written in Vietnamese, lack of leadership from qualified PTs, independence from doctors of traditional medicine, infrastructure, hospital regulations toward EBP, authority to make clinical decisions, money, and minimal information in the PT field.

### Relationships and trends among variables and demographic characterisctics

The attitude toward the need to increase EBP is associated with age, higher qualification, and number of patients per day. Whereas, the attitude toward the statements that the use of EBP is an unreasonable demand on PTs and EBP does not take into account the limitations of PTs’ clinical practice settings was associated with age, years of experience, and number of patients a day, respectively. The knowledge of all the 5 steps of EBP, learning EBP, research methodology, and the competency of reading articles in English showed significant associations related to age and years of experience. In the frequency of EBP use, higher qualification was positively related to integrating the evidence with patient’s preference and therapist’s clinical expertise (step 4), and evaluating outcomes after applying evidence (step 5). Cross-tabs were tested to determine the trend if trend existed among variable. The results show the trends of associations that young participants (20–29 years of age) or those having less than 5 years of experience were more likely to agree that EBP implementation placed unreasonable demands on their tasks compared to those who were 30–39 years of age or had 6 to 14 years of experience. Participants with less than 5 years of experience were more likely to hold certificates as their highest qualification than those who had 10–14 and 15+ years of experience. Participants holding B.Sc. or M.Sc. degrees were more likely to apply step 4 and step 5 than those holding certificates/ 3-year-B.Sc. degrees (Table 6).

**Table 6:** Trends of associations

Attitude toward EBP	Level of years of experience	Odd	95% CI*	*p*-value
The adoption of EBP places an unreasonable demand on physical therapists.	1–5	3.2	1.6–6.2	.001
	6–9			
	1–5	3.3	1.7–6.7	.001
	10–14			
Level of years of age	Level of qualification	Odd	95% CI*	*p*-value
20–29	Certificate/ 3-year B.Sc.	1.7	1.1–2.8	.02
30–39	4-year B.Sc./ M.Sc.			
20–29	Certificate/ 3-year B.Sc.	2.0	1.1–3.7	.03
40–49	4-year B.Sc./ M.Sc.			
20–29	Certificate/ 3-year B.Sc.	2.4	1–5.8	.05
50+	4-year B.Sc./ M.Sc.			
Integrate the found evidence with your expertise for making clinical decisions?	Certificate/ 3-year B.Sc.	2.3	1.2–4.5	.014
	4-year B.Sc./ M.Sc.			
Evaluate the outcome of your applying evidence to your practice?	Certificate/ 3-year B.Sc.	1.9	1.04–3.5	.003
	4-year B.Sc./ M.Sc.			

*CI Confidence Interval

## Discussion

### Attitude, knowledge, and use

Are physical therapists in Viet Nam ready to implement EBP? To a large extent, the answer depends on the age and qualification of therapists. Those with less experience and a lesser qualification do not seem ready. Participants with less experience believe that applying EBP places unreasonable demands on their duties.

The use of all steps of EBP is low, although most of the respondents agreed that EBP is necessary and useful, and that increasing EBP use could keep them current with international healthcare trends [13]. Many participants did not feel confident in appraising professional articles, a result similar to that of a previous study in which research methodology and statistical understanding were also found as barriers [13]. To critique research literature, knowledge of research is necessary, but only half reported that they learned EBP or research in their academic preparation. Therapists with lower qualifications reported less EBP or research preparation. This lack of educational preparation may explain that they did not understand that EBP does take into account patients’ preference or that formatting PICO questions facilitates searching.

Implementing EBP assumes knowledge. The primary tenants of EBP are: practitioners, clinical expertise, knowledge and application of relevant research, and knowledge and attention to patient’s values [1]. A third of our respondents chose a neutral response to the statement that EBP does not take into account patients’ preference. This response reflects the participants’ lack of understanding of EBP, in which patients’ preference is a major pillar. Increased knowledge of EBP seems needed before full implementation is possible.

The incongruous finding of reported knowledge of the 5 steps of EBP, lack of EBP academic preparation and poor English language skills to understand research articles may be due to the bias of a self-administered questionnaire. Participants may also read research articles in a language other than English.

A difficult finding to understand is that most participants reported that they were able to apply evidence (step 4) or review their work after applying the found evidence in their practice (step 5). Although these steps seem to require higher level interpretation, there were fewer reported limitations.

The important role of reading research literature was confirmed in an assessment of Swedish therapists [26]. However, only 25% of those physical therapists reported that they could confidently read English research articles. This finding is consistent with the result of this study and those from countries where English is not the first language [10, 12, 14, 15]. In Viet Nam, to be qualified as a physical therapist, a student must first have had training in a foreign language in middle and high schools. This language could be either English, French, Russian, or Chinese [27]. In professional PT courses, a quarter of general knowledge credits are taught in English [28]. Despite these language requirements, most of our participants reported difficulty in reading the literature, which is primarily written in English. The education for Vietnamese PTs faces a critical English-reading-comprehension challenge that must be addressed immediately. Increasing English competency both written and oral but particular reading and analyzing professional research articles is needed in both academic and continuing education programs.

### Attention to literature

Therapists in Viet Nam do not commonly read professional research literature. The number of respondents reading fewer than 2 articles, using Medline or other database, or researching findings in CDM, almost doubles the number who reported in Colombia [29], the USA [23], and Canada [30]. In contrast, when compared to the frequency of reading 2 to 5 articles, it is nearly two times lower as compared to the Jette’s [23], Akinbo’s [31], and Salbach’s [25] studies. According to the Jetty’s conclusion, the number of 0 to 1 article read per month is too low considering the intent of EBP use to help in clinical decision making. Inadequate English reading competency may be one of the reasons for the infrequent reading of professional articles of PTs in HCMC (Table 3).

### Sources used for CDM

Peers, personal experience, and textbooks were ranked as the top 3 sources used in clinical practice by our participants, a behavior also found from a previous study [32] involving physical therapists and nurses where peers were rated as an easy, ready, and inexpensive source for information-seeking [33]. Textbooks provide good information on background knowledge but are not the source recommended for specific patient related questions about intervention, comparison, and outcome. Rather than relying on current research literature or journal articles, participants preferred to use informal knowledge through interactions with their professional peers or former instructors, a practice which was also reported by others [10, 34]. David [1] recommended that textbooks without update should be dismissed, because that information may not be current. However, most participants used textbooks as a major source of information in CDM instead of research articles, the primary source recommended by EBP. The current practice of using textbooks and peers in CDM should be addressed to improve EBP use by PTs in HCMC. This also may indicate that the information needed by these therapists is background knowledge that is commonly taught in professional education.

### Barriers toward EBP use

Barriers impacting EBP use in Viet Nam are not substantially different from many previous studies in other countries, particularly in developed countries. A list of similar barriers and their sources includes: time, skills, and misperceptions [13]; lack of access to the internet and sources of evidence [35]; inadequate knowledge of statistics and lack of EBP curricular structures [36]; lack of knowledge, skills and interest to transfer research into practice [37]; and lack of language skills in countries where the first language is not English [12, 15]; for example Nigeria, Japan, Colombia, and Viet Nam. In addition, Viet Nam is among the countries with a very high level of workload for PTs, for example, 70% of the participants provided care for more than 11 patients per day. In comparison 54% in the USA [23], 40% in Nigeria [31], 63% in Canada [25] report this high a case load. A high case load was one of the top three barriers in applying EBP in this study. Work overloading affects PTs, patients, and quality of healthcare. This is a critical problem and must be addressed immediately. A high workload seems to be a hindrance to EBP implementation [31] and should be noted as a quantitatively potential factor that impacts the limitation of EBP use.

Although no significant relationship between knowledge of the steps of EBP and qualifications was found, significant relationships between the use of steps 4 and 5 of EBP and qualification were clear. Participants holding certificates were more likely to report that they did not integrate found evidence, or did not evaluate their outcome after applying evidence than those who held B.Sc./M.Sc. degrees. Participants holding certificates and younger therapists were limited in the use EBP. One pillar of EBP is integrating the evidence with one’s clinical expertise. However, this essential component of EBP can only be obtained by good professional education and clinical practice. Young therapists need clinical guidance and should be supported in steps 4 and 5.

### Relationships and trends among variables

The skill in searching for evidence is expected to be strength of young practitioners as reported by many studies [23]. But in these Vietnamese therapists conflicting evidence was found. Younger participants [20–29] were less likely to agree that they were confident or had the ability related to steps 1, 3, 4, and 5 than those who were in older age groups. Without EBP training, young participants might not search successfully and further confirm that young therapists need more support. Compared with older participants (aged 30–49), younger participants [20–29] were likely to have inadequate English reading skill and understanding of research literature. The finding suggests two interpretations: (i) young participants holding physical therapy certificates as their highest qualification did not learn research methodology in their curriculum, which might lead to the high number of young participants having difficulty in understanding research articles; and (ii) PT’s continuing professional education does not focus on understanding and analyzing professional articles. In addition, the significant relationships between ages or years of experience and qualifications show that younger participants or less experienced ones were more likely to hold certificate degrees. This finding highlights that, contrary to the international trend, PT education in Viet Nam is still at the certificate level.

Participants holding certificates as their highest qualification were also more likely than those holding higher qualification to report that they did not integrate found evidence or evaluate the outcome after applying evidence, which might indicate that limited professional training hinders EBP proper implementation.

### Limitations of survey

Due to the nature of the self-administered questionnaire survey, data related to the knowledge of EBP and the frequency of EBP use may have been overestimated. Social effects such as under-reporting, over-reporting, or gaps in EBP knowledge could also be limitations.

### Recommendations

The PT curriculum should be modified to add new essential components such as English technical items, how to read, understand, and appraise research articles, and EBP. Continuing education programming should be developed to assist therapists in improving patient outcomes by applying EBP. Further qualitative studies should be conducted to explore how barriers impact the use of EBP.

## Conclusion

Our results suggest that management and education system in Viet Nam should be changed to ensure that EBP is implemented. Knowledge of EBP principles and steps as well as creating a Positive attitude toward and environment in order to implement EBP are two necessary requirements. Identified barriers need to be addressed including improving the skill of reading English professional articles, providing sufficient time, and facilitating hospital regulation. In order to facilitate the EBP use in Viet Nam, the following suggestions are: (i) job analysis to address the workload; (ii) increase academic and continuing education to improve understanding and applying research, as well as technical and professional terms in English; (iii) require EBP as compulsory credits in theory and practical courses; (iv) implement journal clubs as an effective solution to create a positive environment to discuss and apply evidence, and to decrease the English language barrier.

## Additional file

Additional file 1: Evidence based Questionnaire and Content. (DOCX 25 kb)

## Abbreviations

B.Sc.: Bachelor of Science
CC: Correlation Coefficient
CDM: clinical decision-making
CI: Confidence Interval
EBP: Evidence-based practice
HCMC: Ho Chi Minh City
M.Sc.: Master of Science
PICO: Patient-Intervention-Comparison-Outcome
PTs: Physical therapists
USA: The United States of America
