# Supplementary material for: Are physical therapists in Viet Nam ready to implement evidence-based practice? A survey
Source: BMC Med Educ. 2018 Dec 22;18:317. doi: 10.1186/s12909-018-1428-3 (PMC6303861; doi:10.1186/s12909-018-1428-3)
Supplement: Supplementary file 1 — Evidence based Questionnaire and Content. (DOCX 25 kb) [file 12909_2018_1428_MOESM1_ESM.docx]

**EVIDENCE-BASED PRACTICE QUESTIONNAIRE**

1. **RATE YOUR RESPONSES TO THE FOLLOWING STATEMENTS.**

For the following items, place the mark **X** inside the cell that indicates your response.

| **THIS SECTION REQUIRES ABOUT PERSONAL ATTITUDES TOWARD, USE OF, PERCEIVED BENEFITS AND LIMITATIONS OF EBP.** | **STRONGLY DISAGREE** | **DISAGREE** | **NEUTRAL** | **AGREE** | **STRONGLY AGRREE** |
| --- | --- | --- | --- | --- | --- |
| 1. Application of EBP is necessary in my practice. |  |  |  |  |  |
| 1. Literature and research findings are useful in my day-to-day practice. |  |  |  |  |  |
| 1. I need to increase the use of evidence in my daily practice. |  |  |  |  |  |
| 1. The adoption of EBP places an unreasonable demand on physical therapists. |  |  |  |  |  |
| 1. I am interested in learning or improving the skills necessary to incorporate EBP into my practice. |  |  |  |  |  |
| 1. EBP improves the quality of patient care. |  |  |  |  |  |
| 1. EBP does not take into account the limitation of my clinical practice setting. |  |  |  |  |  |
| 1. EBP helps clinicians remain up-to-date with health care trends. |  |  |  |  |  |
| 1. Strong evidence is lacking to support most of the intervention I use with my patients. |  |  |  |  |  |
| 1. EBP helps me make decisions about patient care. |  |  |  |  |  |
| 1. EBP does not take into account patient preference. |  |  |  |  |  |
| **THIS SECTION INQUIRES ABOUT YOUR KNOWLEDGE TOWARD EBP.** |  |  |  |  |  |
| 1. I am confident to convert information needed into formatted questions using PICO formulation. |  |  |  |  |  |
| 1. I am aware of major information types and resources. |  |  |  |  |  |
| 1. I am able to search the scientific evidence relevant to the formulated question. |  |  |  |  |  |
| 1. I am confident in my ability to critically appraise professional literature. |  |  |  |  |  |
| 1. I am able to apply evidence to my own cases. |  |  |  |  |  |
| 1. I am able to review my own work after applying the found evidence. |  |  |  |  |  |
| 1. I learned the foundation for EBP as part of my vocational/ tertiary education/ continuing education course. |  |  |  |  |  |
| 1. I received research methodology as part of my vocational/ tertiary education/ continuing education course/ self-taught course. |  |  |  |  |  |
| 1. My English language skills are adequate for understanding research articles. |  |  |  |  |  |
| **THIS SECTION INQUIRES ABOUT THE FREQUENCY OF EBP USE** (during 6 months). | **DO NOT USE** | **MONTHLY OR LESS** | **EVERY FORTNIGHT** | **WEEKLY** | **DAILY** |
| 1. How often have you converted information needed into formatted questions by using PICO? |  |  |  |  |  |
| 1. How often have you obtained the evidence relevant to your formulated questions? |  |  |  |  |  |
| 1. How often have you identified the strength and weakness of the evidence you found? |  |  |  |  |  |
| 1. How often have you integrated the found evidence with your expertise for making clinical decisions? |  |  |  |  |  |
| 1. How often have you evaluated the outcome of your applying evidence in your practice? |  |  |  |  |  |
| 1. How often have you shared your ideas and information with colleagues about the outcome of applying evidence? |  |  |  |  |  |
| **This question inquires about the number of research(es) that have been read.** | ≤1 article | 2-5 articles | 6-10 articles | 11-15 articles | 16+ articles |
| 1. I read/review research/literature related to my clinical practice (for a typical month). |  |  |  |  |  |
| **These questions inquire about the frequency of clinical decisions and use of online database.** | ≤1 time | 2-5 times | 6-10 times | 11-15 times | 16+ times |
| 1. I use professional literature and research findings in the process of clinical decision making (for a typical month). |  |  |  |  |  |
| 1. I use MEDLINE or other databases to search for practice-relevant literature/research (for a typical month). |  |  |  |  |  |
| 1. Rank your top 3 choices by placing a number in the appropriate boxes (1 = most important). | **YES** | **NO** | **1** | **2** | **3** |
| Peers |  |  |  |  |  |
| Textbooks |  |  |  |  |  |
| Experts’ opinions |  |  |  |  |  |
| Personal experience |  |  |  |  |  |
| Patient’s preference |  |  |  |  |  |
| Current research literature/ journal article |  |  |  |  |  |
| Original undergraduate training |  |  |  |  |  |
| Information from continuing education |  |  |  |  |  |
| Best current research |  |  |  |  |  |
| Other………………………………………………  …………………………………………………….. |  |  |  |  |  |
| 1. Perceived barriers: Rank your top 3 choices by placing a number in the appropriate boxes (1 = most important). | **YES** | **NO** | **1** | **2** | **3** |
| Insufficient time |  |  |  |  |  |
| Lack of interest |  |  |  |  |  |
| Lack of expertise |  |  |  |  |  |
| Organizational culture |  |  |  |  |  |
| Lack of research skills |  |  |  |  |  |
| Lack of foundation on EBP |  |  |  |  |  |
| Lack of authority to change |  |  |  |  |  |
| Lack of information resources |  |  |  |  |  |
| Lack of manager’s support |  |  |  |  |  |
| Negative attitude toward behavior |  |  |  |  |  |
| Lack of understanding of statistical analysis |  |  |  |  |  |
| Poor ability in foreign language reading skill |  |  |  |  |  |
| Poor ability to critically appraise the literature |  |  |  |  |  |
| Other………………………………………………..  ……………………………………………………… |  |  |  |  |  |

1. **DEMOGRAPHIC INFORMATION**

For the following items, place the mark x inside the box that indicates your response.

1. What is your gender?

🞎 Male 🞎 Female

1. What is your age group?

🞎 20- 29 🞎 30-39 🞎 40-49 🞎 50+

1. How many years have you worked as a physical therapist?

🞎 1-5 🞎 6-9 🞎 10 -14 🞎 15+

1. What is your entry-level degree for PT?

🞎 Vocational high school 🞎 College

🞎 B.Sc.

1. What is your highest degree for PT?

🞎 College 🞎 B.Sc.

🞎 B.Sc. (University transferred) 🞎 Master

🞎 Ph.D.

1. What is your highest degree obtained from another field?

🞎 B.Sc 🞎 Master

🞎 Ph.D. 🞎 None

1. Are you a PT clinical instructor? (If No, please keep to 9.)

🞎 Yes 🞎 No

1. How many years have you been a clinical instructor?

--------- years

1. How many research works have you ever conducted?

-------- research (es)

1. What is the average number of patients you see daily?

🞎 1-3 🞎 4-6 🞎 7-9 🞎 ≥ 10

1. How many hours per week have you spent in taking care of patients?

🞎 ≤ 10 🞎 11-20 🞎 21-30 🞎 31-40 🞎 41+
